# Münchausen Syndrome as an Unusual Cause of Pseudo-resistant Hypertension: A Case Report

**DOI:** 10.1515/med-2019-0094

**Published:** 2019-11-07

**Authors:** Małgorzata Kobusiak-Prokopowicz, Anna Marciniak, Bogdan Tokarczyk, Maria Kała, Jerzy Leszek, Andrzej Mysiak

**Affiliations:** 1 Cardiology Department, Wroclaw Medical University, Borowska 213, 50-556 Wroclaw, Poland; 2 Cardiology Department, Wroclaw Medical University, 50-367 Wroclaw, Poland; 3 Institute of Forensic Research, 31-033 Krakow, Poland; 4 Psychiatry Department, Wroclaw Medical University, 50-367 Wroclaw, Poland

**Keywords:** Müchausen syndrome, Pseudo-resistant hypertension, Iliac arteriovenous fistula, Medication non-adherence

## Abstract

Münchausen syndrome can be characterized by simulated illness, pathological lying and wandering from place to place (the patient typically presents to numerous hospitals). Individuals with elevated blood pressure due to non-adherence to medication have the so-called pseudo-resistant hypertension.

A 45-year-old woman was admitted to hospital on an emergency basis because of a hypertensive crisis. Despite combination antihypertensive treatment, normalization of blood pressure was not achieved and a device to produce a therapeutic arteriovenous fi stula was implanted. Aft er the procedure, a signifi cant increase in pulmonary artery pressure was observed and closure of the fistula was performed by implantation of the stent graft . The suspicion was raised that the patient had not been taking her prescribed medications. Therefore, blood samples were taken and the serum was analyzed for presence of the prescribed drugs (atorvastatin, bisoprolol, chlorthalidone, clonidine, doxazosin, furosemide, nitrendipine, oxazepam and valsartan). The results confirmed suspected failure of the patient to take the prescribed medications.

Münchausen syndrome is usually first suspected when inexplicable laboratory test results are noted. To our knowledge, this is the first reported case of Münchausen syndrome with pseudo-resistant hypertension leading to the implantation of a device to produce a therapeutic arteriovenous fi stula.

## 1 Introduction

Münchausen syndrome can be characterized by three features: simulated illness, either physical or psychiatric; pathological lying (pseudologia phantastica); and wandering from place to place (known as peregrination)—the patient typically presents to numerous hospitals. In this syndrome, the affected person exaggerates or creates illnesses in themselves to gain examination, treatment and sympathy. This disorder is distinct from hypochondriasis and other somatoform disorders [1]. In broad terms, Münchausen syndrome is a type of factitious disorder with predominantly physical signs and symptoms leading the patient to seek hospitalization. In some cases of Münchausen syndrome, people are able to produce symptoms that result in lengthy and costly medical analysis, prolonged hospital stays and unnecessary operations [2,3]. The diagnosis of Münchausen syndrome is reserved for the most severe form as no effective treatments have been identified through well-conducted studies.

Patients with Münchausen syndrome often present with dramatic symptoms of somatic diseases; however, the doctor will only begin to consider the psychological basis of the disease after a multi-stage diagnosis. This happens when, contrary to physical examination, there is little or no organ damage [4]. In the case of hypertension, the diagnosis of which is based largely on self-measurement of blood pressure, verification of the diagnosis of refractory hypertension is particularly difficult.

Resistant hypertension is defined as blood pressure remaining above the goal blood pressure despite the use of three or more antihypertensive medications at maximally tolerated doses. Resistant hypertension is divided into true resistant hypertension, controlled resistant hypertension and pseudo-resistant hypertension [5]. Individuals with elevated blood pressure due to white-coat hypertension, improper measurement or medication non-adherence have the so-called pseudo-resistant hypertension [6]. Münchausen syndrome has been recently included in the list of causes of pseudo-resistant hypertension [7].

## 2 Case presentation

A 45-year-old woman who had been treated for hypertension for several years, with visual impairment due to retinopathy and left hemiparesis after ischemic stroke, was admitted to the cardiology department on an emergency basis because of a hypertensive crisis (RR 230/130 mmHg).

In the interview, the patient reported headaches, dyspnea at rest, decreased tolerance of physical effort and pain on the left side of the body (from the scapula and chest, to the left subcostal region). She denied typical exercise-induced angina or syncopes. The ECG did not record features of myocardial ischemia. Echocardiography showed no left ventricular systolic dysfunction and the EF was 55%; however, hypertensive myocardial damage was observed in the form of left ventricular diastolic dysfunction, ventricular septal hypertrophy and left atrial enlargement.

According to the medical records provided, the patient had been hospitalized repeatedly due to resistant hypertension. She was admitted to hospital for the first time in December 2012 to a neurological ward due to cerebral ischemic stroke complicated by left hemiparesis. Despite being given combination antihypertensive treatment, normalization of blood pressure was not achieved. The patient was then referred to a department of internal medicine, where a broad assessment of secondary causes of hypertension was performed, including thoracic aorta angio-CT (radiological features of aortic dissection were excluded), abdominal CT (multiple vascularization of both kidneys was revealed) and hormone testing (blood levels of TSH, ACTH, cortisol and renin, and daily urinary excretion of methoxy-catecholamines and vanillylmandelic acid, were within normal limits). A polysomnographic study excluded the presence of a breathing disorder during sleep. In addition, the patient was assessed for complications of hypertension, and microalbuminuria was diagnosed. During hospitalization, antihypertensive therapy was repeatedly modified to obtain a reduction in blood pressure to 150/100 mmHg.

Due to a long history of resistant hypertension, the patient was hospitalized repeatedly in the years 2013–2014 in centers specializing in the diagnosis and treatment of hypertension. In September 2013 abdominal ultrasonography was performed, with no change in kidney or adrenal imaging; however, in the Doppler study, multiple renal vascularization was confirmed (three renal arteries on the right side, two renal arteries on the left), with no signs of stenosis. The patient therefore did not qualify for renal artery denervation. Because of persistently high blood pressure despite the use of multiple pharmacological and non-pharmacological treatment options (including diet and weight reduction), the patient qualified for surgical treatment of hypertension—i.e. creation of a therapeutic iliac arteriovenous fistula.

Creation of an iliac arteriovenous fistula was planned to be performed in December 2013; however, the intervention was postponed due to high blood pressure in pulmonary capillaries identified during right heart catheterization. In January 2014, implantation of a device to produce a therapeutic arteriovenous fistula between the iliac artery and the external iliac vein was performed without complications.

About 4 weeks after the procedure, the patient was admitted to the cardiology department due to exercise dyspnea with minimal physical effort that had been increasing over the past 2 weeks. Dyspnea was accompanied by persistent coughing, which increased when lying down, and the feeling of palpitations. Coronary angio-CT did not show any atherosclerotic lesions in the epicardial arteries, but a deep vascular bridge in the 7^th^ LAD segment (intramuscular course of the artery) was shown. Considering the temporal relationship between the onset of symptoms and the date of controlled creation of the arteriovenous fistula, right heart catheterization was performed, showing non-capillary pulmonary hypertension (mPAP 44 mmHg, PCW 30 mmHg), hyperkinetic circulation (cardiac output 10.3 l/min) and normal pulmonary resistance (1.4 Wood units). Diuretics were administered intravenously, resulting in only a slight reduction in symptoms, primarily relief of dyspnea when lying down and cough.

After exclusion of pulmonary embolism on angio-CT and reconfirmation of the significant increase in pulmonary artery and capillary pressure during right heart catheterization in comparison to imaging before fistula implantation, closure of the therapeutic iliac arteriovenous fistula was performed in March 2014 by implantation of the stent graft in the right external iliac artery. After the procedure, the symptoms of circulatory failure were reversed, while blood pressure increased again. Treatment with seven antihypertensive drugs was continued, and blood pressure values ranged from 160–220/90–120 mmHg.

In June 2015, during hospitalization at the ICCU due to another increase in blood pressure, the suspicion was raised that the patient did not take prescribed medications. To verify this hypothesis, blood samples collected from the patient on subsequent days (i.e. on a day the patient was taking the medications on her own and on the next day, when the patient was supervised taking the medications) were sent to the Institute of Forensic Research for analysis. Blood serum was analyzed by HPLC-DAD, LC-MS and LC–MS/MS for the presence of prescribed medications (atorvastatin, bisoprolol, chlorthalidone, clonidine, doxasosin, furosemide, nitrendipine, oxazepam and valsartan).

In the serum taken on the day on which the patient was taking the drugs by herself, only oxazepam and atorvastatin were found. In the serum taken on the day on which the patient took the drugs under supervision, all prescribed medications were found. Determined concentrations of the prescribed drugs and their therapeutic ranges are listed in Table 1.

**Table 1:** Serum prescribed drugs concentrations, their therapeutic ranges and limits of quantitation of the methods used

Prescribed drug	Concentration [ng/ml]		
	determined	therapeutic range	limit of quantitation
Herself taking			
Atorvastatin	<5	2.5 – 35.3	1
Oxazepam	25	100 – 1400	1
Controlled taking			
Atorvastatin	14	2.5 – 35.3	1
Bisoprolol	94	10 – 60	1
Chlorthalidone	375	200 – 1400	1
Doxazosin	58	80 – 150	1
Furosemide	500	200 – 5000	400
Clonidine	1.6	0.3 – 1.5	1
Oxazepam	42	100 – 1400	1
Nitrendipine	positive*	10 – 15	N/A
Valsartan	positive *	1640 – 3460	N/A

* - qualitative result – lack of standard; N/A – not available

The results of the analyses confirmed suspected failure of the patient to take the prescribed medications. In the next 24 hours, during the controlled drug taking, the RR was reduced to 90/60 mmHg, and the antihypertensive doses were therefore reduced to achieve a target blood pressure of <140/90 mmHg.

**Ethical approval:** The research related to human use has been complied with all the relevant national regulations, institutional policies and in accordance the tenets of the Helsinki Declaration.

**Consent for publication:** Informed verbal consent written down in the case record was obtained from the patient for the publication of this Case Report.

## 3 Discussion

Resistant hypertension is increasingly common in clinical practice. Individuals with true resistant hypertension were found to have a 2.94 times greater cardiovascular risk than those with controlled hypertension [8]. The strictest definition of refractory hypertension is based on the inability to control high blood pressure with use of five or more different classes of antihypertensive agents, including a long-acting thiazide-type diuretic and a mineralocorticoid receptor antagonist [9].

One of the challenges of establishing the prevalence of true resistant hypertension is excluding participants from the test population with pseudo-resistant hypertension, particularly patients with psychic disorders [10]. Clinicians should especially exclude pseudo-resistant hypertension that results from non-adherence to medications [11]. The study by Jung et al., which was designed to identify the degree of medication adherence in patients with resistant hypertension, showed that 53% of patients with uncontrolled hypertension were non-adherent to prescribed drugs when assessed by toxicological urine analysis. Among those who were non-adherent, 30% were taking no antihypertensive medication [12]. Furthermore, Daugherty et al. found that 12.4% of patients with uncontrolled resistant hypertension were non-adherent to their medical regimens, as defined by pharmacy refill rates [13]. Poor adherence to antihypertensive drug treatment is therefore a common cause of pseudo-resistance among patients with apparent resistant hypertension. Pseudo-resistant hypertension would need to be excluded by an established method of assessing adequate medication adherence with standardized measurements and 24-h ambulatory blood pressure monitoring.

Taking into account the complete medical history of our patient, we can confidently diagnose a factitious disorder, described as a condition in which the patient feigns or produces symptoms and signs with the aim of adopting the sick role. The factitious disorder with predominantly physical signs and symptoms, often referred to as Münchausen syndrome, is often combined with personality disorders. Patients usually have good knowledge about the disorders they feign, including their symptoms, diagnostic criteria and treatment, and can therefore be very convincing and hard to identify, even for experienced clinicians. They often undergo many surgical operations and procedures and have surgical scars. Presented symptoms may suggest a disorder related to any organ system, or even mental illness. Patients may intentionally intoxicate or cause physical damage to themselves, take unnecessary drugs or contaminate laboratory samples.

Patients with a factitious disorder are often described by medical personnel as contradictory, demanding and abusive. They question negative results of analyses and examinations, demand further tests and accuse clinicians of incompetence. After being diagnosed, they usually deny their disorder, do not undertake proper treatment, and often sign out from the hospital and then go to another one, sometimes in another city.

The case of our patient teaches us that detailed history and physical examination, including prompt laboratory evaluation, should be mandatory to ensure early diagnosis and management. This kind of disorder is usually suspected when inexplicable laboratory results are noted in the course of a prolonged clinical investigation as in our case [14].

Pessina et al. presented the case of a young man who underwent transcatheter renal denervation due to unstable blood pressure. The procedure did not normalize the blood pressure, so the patient was admitted to the endocrinology department, where pheochromocytoma was diagnosed. He underwent left adrenalectomy, but no adrenal pathology was confirmed on histopathological examination, leading the authors to consider the possibility of Munchausen syndrome [7]. In a commentary on the article, Zorzi et al. referred to an analogous situation, emphasizing that even young patients may present with Münchausen syndrome [15].

We searched the literature for cases of Munchausen syndrome similar to this one (in women aged 45-50, working in healthcare services). Spitzer et al. presented the case of a 46-year old female nursing sister with hypertension, in whom venous blood tests from various areas showed significantly raised concentrations of adrenaline and noradrenaline. First, the right adrenal gland was removed, but this turned out to be completely normal on histopathological examination. A 6-month period of stabilization of arterial pressure was achieved, after which uncontrolled pressure peaks returned. Comprehensive, in-depth diagnostics for refractory hypertension were carried out and no other pathology was found [16].

Being aware of the occurrence of Münchausen syndrome in cardiac patients and reviewing the literature of cases in which the question of the final diagnosis was left unanswered, one can get the impression that the described patients would qualify for in-depth psychiatric diagnosis.

For example, in a study by Chiarito et al., a 47-year-old woman underwent a first transcatheter renal denervation, which did not result in the long-term normalization of arterial pressure. The team of doctors, however, decided to perform another procedure of denervation, which also did not improve blood pressure control [17]. In such situations, the lack of therapeutic success creates considerable discomfort for the medical staff, which can be used by a patient with Münchausen syndrome to enforce the performance of further diagnostic and therapeutic procedures.

## 4 Conclusions

Münchausen syndrome is a challenging condition that often goes undiagnosed by physicians [18]. To our knowledge, this is the first reported case of pseudo-resistant hypertension leading to implantation of a device to produce a therapeutic arteriovenous fistula between the iliac artery and external iliac vein.

## 5 Take home messages

1. The first step to identifying patients with Münchausen syndrome should be to complete a detailed history and physical examination, using repetition to identify biopsychosocial inconsistencies. Consistently reported clues in Münchausen syndrome include dramatic presentation, inconsistent histories, recurrent illnesses that worsen or change after appropriate treatment and attempts to dictate treatment.
2. If Münchausen syndrome is suspected, a psychiatric evaluation should be done as early as possible. Patients with no other psychiatric illness have a better chance of full recovery if diagnosed early.
3. Münchausen syndrome is a recently identified new cause of pseudo-resistant hypertension that should be kept in mind.

## List of abbreviations

ACTH -: adrenocorticotropic hormone
CT -: computed tomography
ECG -: electrocardiogram
EF -: ejection fraction
LAD -: left anterior descending
ICCU -: intensive cardiac care unit
HPLC-DAD -: high-performance liquid chromatography coupled with diode-array detection
LC-MS -: liquid chromatography-mass spectrometry
LC-MS/MS -: liquid chromatography–tandem mass spectrometry
mPAP -: mean pulmonary artery pressure
PCW -: pulmonary capillary wedge pressure
TSH -: thyroid stimulating hormone

## References

[1] Mehta N., Khan J (2007). Cardiac Munchausen Syndrome. Chest.

[2] Yates G.P., Feldman M.D (2016). Factitious disorder: A systemic review of 455 cases in the professional literature. Gen Hosp Psychiatry.

[3] Scott A., Simpson M.D., Pasic J (2016). The peregrinathy psychiatric patient in the emergency department. West J Emerg Med.

[4] Ponte P.H., Matas L., Cadafalch J., Arroyo J.A (2016). Resistant hypertension and no organ damage: a new case of munchausen syndrome. Med Clin (Barc.).

[5] Judd E., Calhoun D.A (2014). Apparent and true resistant hypertension: definition, prevalence and outcomes. J Hum Hypertens.

[6] Sarafidis P.A., Georgianos P., Bakris G.L. (2013). Resistant hypertension—its identification and epidemiology. Nat Rev Nephrol.

[7] Pessina A.C., Bisogni V., Fassina A., Rossi G.P (2013). Munchausen syndrome: a novel cause of drug resistant hypertension. J Hypertens.

[8] Pierdomenico S.D., Lapenna D., Bucci A., Di Tommaso R., Di Mascio R., Manente B.M., Mezzetti A (2005). Cardiovascular outcome in treated hypertensive patients with responder, masked, false resistant, and true resistant hypertension. Am J Hypertens.

[9] Dudenbostel T., Siddiqui M., Oparil S., Calhoun DA (2016). Refractory hypertension: a novel phenotype of antihypertensive treatment failure. Hypertension.

[10] Zakka K., Bitar M., Lakkis B., Koubar S.H (2017). Induced vomiting for attention seeking and secondary gain: An unusual cause of pseudo-resistant hypertension. JRSM Open.

[11] Vongpatanasin W (2014). Resistant Hypertension A Review of Diagnosis and Management. JAMA.

[12] Jung O., Gechter J.L., Wunder C., Paulke A., Bartel C., Geiger H. (2013). Resistant hypertension? Assessment of adherence by toxicological urine analysis. J Hypertens.

[13] Daugherty S.L., Powers J.D., Magid D.J., Tavel H.M., Masoudi F.A., Margolis K.L. (2012). Incidence and prognosis of resistant hypertension in hypertensive patients. Circulation.

[14] Kenedi Ch.A., Shirey K.G., Hoffa M., Zanga J., Lee J.C., Harrison J.D., Preud’homme XA (2011). Laboratory diagnosis of factitious disorder: a systematic review of tools useful in the diagnosis of Munchausen’s syndrome. N Z Med J.

[15] Zorzi F., Oliveri O., Pizzolo F (2014). Comment on “Munchausen syndrome: a novel cause of drug-resistant hypertension.”. J Hypertension.

[16] Spitzer D., Bongartz D., Ittel T.H., Siebert H.G (1998). Simulation of a pheocromocytoma- Munchausen syndrome. Eur J Med Res.

[17] Chiarito M., Scotti A., A Pivato, C., Cottone G., Ballarotto C., Godino C., Margonato A (2017). A complicated case of resistant hypertension. Acta Med Iran.

[18] Sousa Filho, D., Kanomata E.Y., Feldman R.J., Maluf Neto, A (2017). Munchausen syndrome and Munchausen syndrome by proxy: a narrative review. Einstein (Sao Paulo).

